# Synergistic action of synthetic peptides and amphotericin B causes disruption of the plasma membrane and cell wall in *Candida albicans*

**DOI:** 10.1042/BSR20232075

**Published:** 2024-04-12

**Authors:** Thayna A.M. Souza, Erica O. Mello, Gabriel B. Taveira, Felipe F. Moreira, Sergio Henrique Seabra, André O. Carvalho, Valdirene M. Gomes

**Affiliations:** 1 Laboratório de Fisiologia e Bioquímica de Microrganismos, Centro de Biociências e Biotecnologia, Universidade Estadual do Norte Fluminense Darcy Ribeiro, CEP: 28013-602, Campos dos Goytacazes, RJ, Brazil; 2 Laboratório de Biologia Celular e Tecidual, Centro de Biociências e Biotecnologia, Universidade Estadual do Norte Fluminense Darcy Ribeiro (UENF), Campos dos Goytacazes, RJ, Brazil

**Keywords:** Antimicrobial peptide, Bioactive molecules, Cell Membrane, Peptide-based therapeutics, Yeasts

## Abstract

The objective of this work was to evaluate the combination of synthetic peptides based on the γ-core motif of defensin *Pv*D_1_ with amphotericin B (AmB) at different concentrations against *Candida albicans*. We applied the checkerboard assay using different concentrations of the commercial drug AmB and the synthetic peptides γ_31-45_*Pv*D_1_^++^ and γ_33-41_*Pv*D_1_^++^ against *C. albicans*, aiming to find combinations with synergistic interactions. Between these two interactions involving γ_31-45_*Pv*D_1_^++^ and AmB, an additive effect was observed. One such interaction occurred at concentrations of 0.009 µM of peptide γ_31-45_*Pv*D_1_^++^ and 13.23 µM of AmB and another condition of 0.019 µM of peptide γ_31-45_*Pv*D_1_^++^ and 6.61 µM of AmB. The other two concentrations of the interaction showed a synergistic effect in the combination of synthetic peptide γ_31-45_*Pv*D_1_^++^ and AmB, where the concentrations were 1.40 µM peptide γ_31-45_*Pv*D_1_^++^ and 0.004 µM AmB and 0.70 µM γ_31-45_*Pv*D_1_^++^ peptide and 0.002 µM AmB. We proceeded with analysis of the mechanism of action involving synergistic effects. This examination unveiled a range of impactful outcomes, including the impairment of mitochondrial functionality, compromise of cell wall integrity, DNA degradation, and a consequential decline in cell viability. We also observed that both synergistic combinations were capable of causing damage to the plasma membrane and cell wall, causing leakage of intracellular components. This discovery demonstrates for the first time that the synergistic combinations found between the synthetic peptide γ_31-45_*Pv*D_1_^++^ and AmB have an antifungal effect against *C. albicans*, acting on the integrity of the plasma membrane and cell wall.

## Introduction

In recent decades, a drastic increase in systemic fungal infections has been observed, which has led researchers worldwide to seek the development of new effective drugs [1]. Commercial antifungals, like amphotericin B (AmB), is considered the gold standard due to its high efficacy in different types of infections, despite its severe side effects [2].

Polyenes belong to a category of antibiotics that are readily accessible on the market and are primarily employed in the treatment of fungal ailments. Notable members of this group include nystatin, natamycin, and AmB. Among these, AmB stands out as the most potent in terms of toxicity [3]. The ongoing evaluation of polyenes in clinical applications, particularly AmB, stems from certain drawbacks associated with their use as antifungal agents. The mechanism of their action entails binding to ergosterol, resulting in the creation of pores within the cell membrane of fungi. This disruption ultimately leads to oxidative damage and membrane rupture. Despite its status as a potent broad-spectrum fungicidal treatment characterized by a low occurrence of microbial resistance, the clinical utilization of AmB is still restricted [4,5].

AmB boasts an amphipathic character and is distinguished by its notable water solubility. Its conventional administration involves a formulation known as AmB deoxycholate (D-AMB), which uniquely forms micelles upon introduction into aqueous solutions. Nonetheless, concerns regarding potential nephrotoxicity and cardiotoxicity associated with D-AMB have ignited substantial interest within the scientific community. As a result, considerable efforts have been channeled into exploring synergistic combinations involving other compounds in this domain, as noted by Carolus and Cavassin [3,6]. Antimicrobial peptides (AMPs) naturally occur in nature and can also be engineered through rational synthesis. They exhibit broad-ranging antimicrobial activity and are particularly effective against many strains of fungi. Several research efforts have focused on investigating the synergistic combination of these peptides with commercial antifungals, with the aim of creating new therapeutic agents that not only increase efficacy but also decrease the occurrence of adverse effects [2,7,8].

The yeasts of the genus *Candida*, mainly *C. albicans*, are yeasts that interact with the host in the commensal form and can be isolated from the gastrointestinal tract and oral and vaginal mucosa of many, if not all, healthy individuals. As one of the most widespread fungi in the human microbiome, *C. albicans* is one of the main causes of fungal infections, with invasive candidiasis being the cause of the high mortality rate, which varies from 46 to 75%. The progression of *C. albicans* pathogenesis is driven by alterations in the morphology of yeast cells, transitioning into hyphae (or pseudohyphae), accompanied by an augmented generation of virulence factors during infection [9].

Drug interactions have been a path that researchers have pioneered in the search for new, more effective and less toxic drugs. The synergistic combination of caspofungin (CAS) and fluconazole was effective in decreasing *C. glabrata* resistance, and other interactions have been described [10–12]. Combinatorial therapy has emerged as a pivotal strategy in the quest for novel antifungal agents, aiming to discover fresh therapeutic modalities. Among these, AMPs present a significant alternative, highlighting their ability to yield bactericidal and antifungal effects, among other benefits, when employed in conjunction with other treatments [12–14].

AMPs have been known since the 1940s. However, in the early 1980s, their central role in the innate immunity of different organisms was revealed, such as insect cecropin, mammalian defensins and magainin in amphibians [15]. Its broad antimicrobial activity has increased interest in these molecules. In addition, AMPs have some characteristics that make them excellent therapeutic agents, such as broad antimicrobial activity, making this molecule active against different microorganisms. Its small size and high stability in insulting environments, and its ease of engineering, peptides by molecular biology or even chemical synthesis [16]. This last technique has the advantage of allowing the incorporation of uncommon amino acids, such as nonproteinogenic or D-enantiomers, into AMP primary structures, improving their activity, specificity and stability, decreasing their toxicity and protecting them from proteolytic degradation in the target organism [17].

Synthetic antimicrobial peptides thus emerge as an option for combination with already commercial drugs in the search to increase their activity and reduce side effects. The plant family of defensins are cationic peptides with a characteristic domain of eight cysteine residues forming four disulfide bonds. They are part of the important components produced in the plant defense system and are located on the periphery of different organs, such as seeds and fruits [18,19]. Synthetic peptides derived from the primary structure of plant defensins have been extensively studied, with particular emphasis on the γ-core motif region. This region is of significant interest due to its crucial role in the antimicrobial activity of AMPs [19,20]. The γ-core motif has emerged as an attractive site for targeted modifications in plant defensins, as certain defensins have demonstrated activity in this specific region. The γ-core motif in plant defensins is represented by the formula NH_2_–[X_1-3_] - [GXC] - [X_3-9_] - [C]-COOH, with X denoting any amino acid [20].

In 2019 [21] Mello et al. designed four new peptides inspired by the γ-core region of *Phaseolus vulgaris* defensin (*Pv*D_1_). The peptides synthesized were γ_31-45_*Pv*D_1_ (RSGRARDDFRAWATK), consisting of 15 amino acids from Arg31 to Lys45, which includes parts of the β2 and β3 sheets. The modified version, γ_31-45_*Pv*D_1_^++^, was created to enhance the positive charge by substituting two aspartic acid residues at positions 37 and 38 with arginine (RSGRARRRFRAWATK). γ_31-45_*Pv*D_1_ and γ_31-45_*Pv*D_1_^++^ had net charges of +3 and +7 and molecular masses of 1792.99 and 1875.19 Da, respectively. Additionally, two other peptides were synthesized, each comprising 9 residues. The first one, γ_33-41_*Pv*D_1_ (GRARDDFRA), corresponds to the native γ-core motif from Gly33 to Cys41 and exhibited a charge of +1 and a molecular mass of 1063.14 Da. The second one, γ_33-41_*Pv*D_1_^++^, is a modified version with increased positive charge achieved by replacing the two aspartic acid residues with arginine (GRARRRFRA), showing a charge of +5 and a molecular mass of 1145.34 Da. Remarkably, the peptide γ_33-41_*Pv*D_1_^++^ demonstrated the most toxic effects on the yeast *C. buinensis*, suggesting that this peptide triggers cell death via apoptosis.

In ththe present study, our objective was to evaluate the combination of synthetic peptides based on the γ-core motif of defensin *Pv*D_1_ (γ_33-41_*Pv*D_1_^++^ and γ_31-45_*Pv*D_1_^++^) with AmB against *C. albicans* in the search for a synergistic concentration to contribute to the development of new drugs.

## Materials and methods

### Microorganisms

The yeast *Candida albicans* was obtained from Departamento de Biologia, Universidade Federal do Ceará, Fortaleza, Brazil. Yeast was maintained on Sabouraud 2% glucose ágar (Merck) at the Laboratório de Fisiologia e Bioquímica de Microrganismos, Centro de Biociências e Biotecnologia, UENF, Campos dos Goytacazes, Rio de Janeiro, Brazil.

### Synthetic peptides

This study employed two peptides designed by Mello et al. [21] The first peptide, γ_31-45_*Pv*D_1_^++^, consisted of 15 amino acid residues with a charge of +7 (RSGRARRRFRAWATK). The second peptide, γ_33-41_*Pv*D_1_^++^, was composed of 9 amino acid residues with a charge of +5 (GRARRRFRA). AminoTech (São Paulo, Brazil) performed the peptide synthesis and conducted quality and purity analyzes (≥ 95%) using reversed-phase high-performance liquid chromatography (RP-HPLC) and mass spectrometry. The synthetic peptides were solubilized in water and utilized in all *in vitro* assays.

### Antifungal assay with synthetic peptides

For yeasts, aliquots were taken from plates containing grown colonies and placed in new Petri dishes containing Sabouraud agar (10 g/L peptone, 2 g/L glucose, 20 g/L agar) (Merck) striations on the middle. These new plates were kept in an oven at 30°C for 24 h. After growth, with a Drigalski loop, the cells were removed and homogenized in 10 ml of Sabouraud broth (10 g/L peptone, 2 g/L glucose) (Merck) for quantification in a Neubauer chamber (LaborOptik) with the aid of an optical microscope (Axiovison A2, Zeiss). Subsequently, the yeast cells (1 × 10^4^ cell ml^−1^) were incubated in Sabouraud broth containing preestablished concentrations of the synthetic peptides and AmB, which ranged from 1.56 to 200 μM. The assay was performed in cell culture microplates (96 wells) at 30°C for 24 h. Cell growth was determined by optical density, monitored at 24 h in a microplate reader at a wavelength of 620 nm. Each test will be performed in triplicate. The entire procedure will be performed under aseptic conditions in a laminar flow, according to the methodology adapted [22]. The MIC_100_ was determined as the minimum inhibitory concentration in μM required to completely inhibit fungal growth, with visual interpretation used for assessment. On the other hand, the MIC_50_ represents the peptide concentration in μM that resulted in 50% inhibition of fungal growth, and it was estimated using nonlinear regression analysis.

### Checkerboard assay

For the evaluation of the interaction between the different concentrations of each compound, the fractional inhibitory concentration index (FIC) was calculated according to the following formula:

A/MIC A +B/MIC B = FIC A +FIC B = FIC index

where A and B are the MICs of each drug used individually. The FIC index value is then used to categorize the interaction of the two antifungals used. The combination is considered synergistic when the FIC index value is < 0.5, showing that the combination of the compounds increases the inhibitory activity of one of the compounds. When the FIC index value is between 0.5 and 4, the combination is considered indifferent or additive, meaning that the combination of compounds does not increase the inhibitory activity or slowly increases the activity due to the additive effect of both combinations. An FIC index > 4 categorizes antagonism, which occurs when the combination of compounds decreases the activity of the compounds. To evaluate the combinatorial effect of synthetic peptides and AmB against *C. albicans*, the serial microdilution technique for the checkerboard assay was performed according to the methodology described [23]. One hundred microliters of standardized *Candida* suspensions (1 × 10^4^ cell ml^−1^), which were further diluted one-fold, were added to each well, and plates were incubated at 30°C for 24 h. The final concentration ranged from 13.23 to 0.82 μM for peptides γ_33-41_*Pv*D_1_^++^, 11.25 to 0.70 μM for γ_31-45_*Pv*D_1_^++^, and 0.019 to 0.001 μM for AmB. Cell growth was determined by optical density in a microplate reader at a wavelength of 620 nm (EZ Read 400, Biochrom).

### Yeast viability assay

To check whether the inhibition of yeast growth was caused by fungicidal or fungistatic activity, the different groups of yeast cells (treatment and control) were washed and diluted 1,000-fold. Yeast cells were quantified in a Neubauer chamber and an aliquot with 1 × 10^2^ cells ml^−1^ was prepared and spread with a Drigalski spatula on the surface of a Petri dish containing Sabouraud agar and grown at 30°C for 36 h. At the end of this period, the colony-forming units were counted, and the Petri dishes were photographed.^13^ The experiments were carried out in triplicate, and the results are shown assuming that the control represents 100% viability.

### Mitochondrial function assay

Mitochondrial functionality was evaluated by light fluorescence microscopy using the fluorescent probe MitoTracker Red FM (Thermo Fisher). After 24 h of incubation, cells from *C. albicans* were incubated with 200 nM MitoTracker Red FM for 30 min at 25°C. Then, the cells were observed under an optical microscope (Axioplan. A2, Zeiss) attached to an AxioCAM MRc5 camera (Zeiss), and the images were analyzed by Axiovision version 4.0 software (Zeiss) equipped with a 581 nm excitation filter and a 644 nm emission filter. A control with cells heated in the presence of 300 μM acetic acid for 15 min was performed. The experiment was repeated three times.

### Detection of chromatin condensation in *Candida albicans*

Nuclear staining with 4',6-diamidino-2-phenylindole (DAPI, Sigma‒Aldrich) was performed as previously described [24]. The synergistic conditions and controls were transferred to a 96-well cell culture plate and incubated for 24 h. A higher cell density was used in this assay to allow microscopic visualization of cells. For nuclear staining, after incubation, the cells were washed with PBS, incubated with 4 μg/ml DAPI in PBS for 10 min, rinsed three times with PBS and then visualized under a DIC epifluorescence microscope (Axio Imager. A2, Zeiss) equipped with a fluorescence filter set (excitation 365 nm; emission 397 nm). The experiment was repeated three times.

### Detection of wall integrity in *Candida albicans*

To assess the integrity of the cell wall, we employed the fluorescent probe Calcofluor White (Sigma‒Aldrich) in our analysis. After subjecting the samples to synergistic conditions for 24 h, a 100 µl portion of the solution was extracted. Subsequently, this portion was mixed with 10 µl of Calcofluor White and incubated for 5 min at 30°C in the dark. Following the aforementioned duration, the cells were examined using a light microscope (Axioplan. A2, Zeiss) coupled with an AxioCAM MRc5 camera (Zeiss). The acquired images were subsequently processed using Axiovision version 4.0 software (Zeiss), which was equipped with a fluorescence filter set (excitation 365 nm; emission 397 nm). To ensure reliable results, the experiment was replicated three times.

### Ultrastructure analysis

For analysis of the ultrastructure of *C. albicans* cells, the synergistic conditions of 1.40 µM peptides γ_31-45_*Pv*D_1_^++^ and 0.004 µM AmB or 0.70 µM peptides γ_31-45_*Pv*D_1_^++^ and 0.002 µM AmB were fixed for 1 h in a solution containing 2.5% glutaraldehyde and 4% freshly prepared formaldehyde in sodium cacodylate buffer 0.1 mol L^−1^, pH 7.4. After these procedures, the cells were washed twice with phosphate buffered saline (PBS) for 10 min and postfixed for 1 h in the dark with a solution containing 1% osmium tetroxide (OsO_4_) and 1.6% ferrocyanide in 0.1 M sodium cacodylate buffer. Subsequently, the cells were washed in the same buffer, dehydrated in acetone (30%, 50%, 70%, 90%, 100% and 100% super dry) and embedded in Epon [25]. Ultrathin sections were stained with uranyl acetate and lead citrate and observed under a Jeol JEM 1400 Plus transmission electron microscope.

### Statistical analysis

Antimicrobial assays were performed in triplicate and repeated three times. Graphs were plotted as the means with standard deviation from an independent assay for antimicrobial assays. The data obtained in the tests were statistically tested by one-way ANOVA, where *p*<0.05 and p<0.01 were considered significant, using GraphPad Prism 8 software.

## Results

### Effect of synthetic peptides and amphotericin B on *Candida albicans*

**Figure 1:**
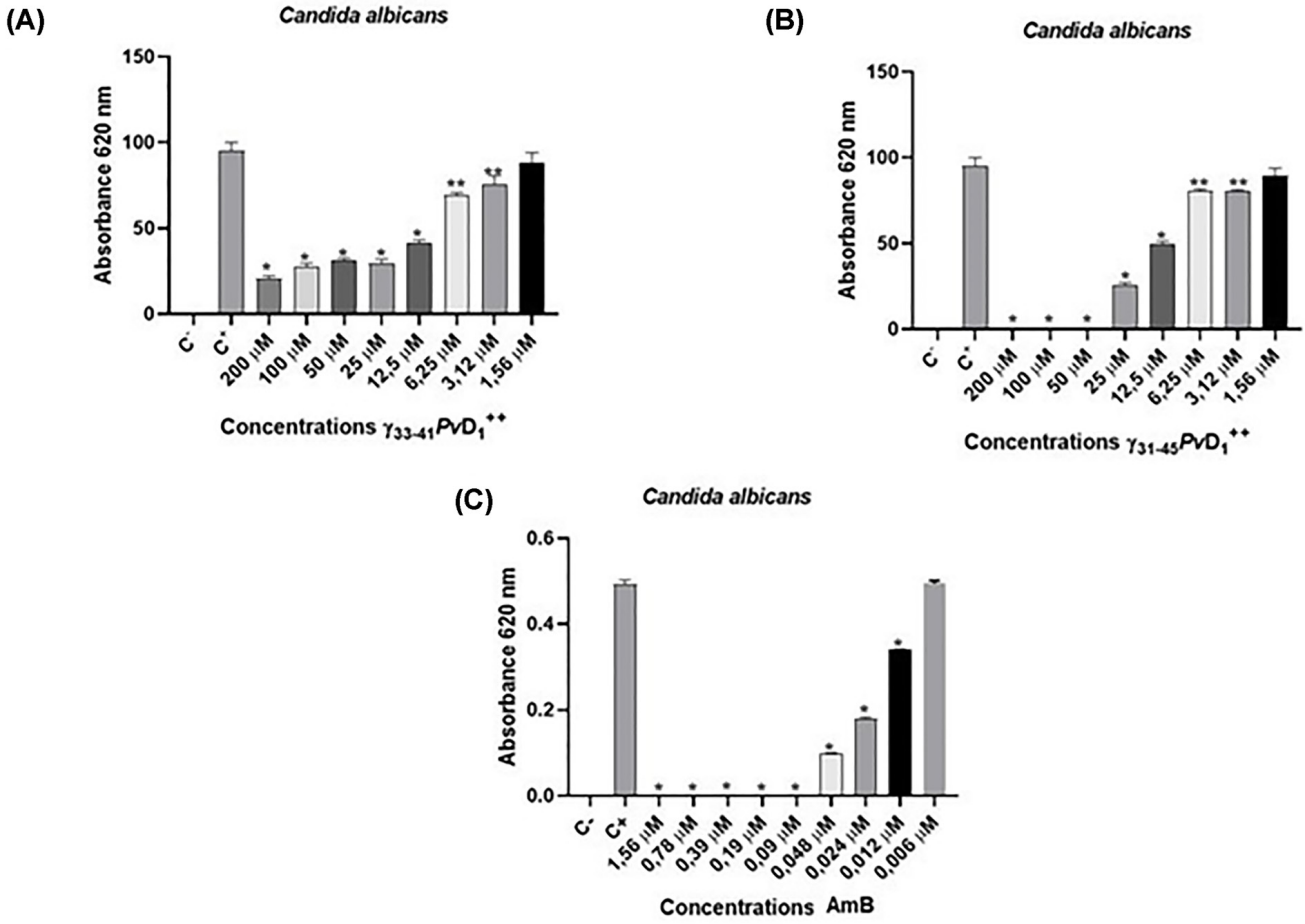
Effect of synthetic peptides and AmB on *C. albicans* cells Growth of *C. albicans* incubated for 24 h in the presence of γ_33-41_*Pv*D_1_^++^ (**A**), γ_31-45_*Pv*D_1_^++^ (**B**) and AmB (**C**). All experiments were performed in triplicate; *p<0.05; **p<0.01.

Figure 1 shows the antifungal activity of the peptides γ_31-45_*Pv*D_1_^++^, γ_33-41_*Pv*D_1_^++^ and AmB. This figure shows the inhibition profile of the molecules against the growth of *C. albicans*. When performing the methodology to find the minimum inhibitory concentration (MIC) of the molecules used that inhibit 50% of cell growth, the results showed that 11.25 µM γ_31-45_*Pv*D_1_^++^, 13.23 µM γ_33-41_*Pv*D_1_^++^ and 0.09 µM and 0.019 µM AmB were able to inhibit 50% of the cell growth of *C. albicans* yeast, while 0.9 µM AmB was able to inhibit 100% of the cell growth (Table 1).

**Table 1:** Minimum inhibitory concentration (µM) of synthetic peptides and AmB

*Candida albicans*			
AmB MIC_100_ (µM)	AmB MIC_50_ (µM)	γ_33-41_*Pv*D_1_^++^ MIC_50_ (µM)	γ_31-45_*Pv*D_1_^++^ MIC_50_ (µM)
0.09	0.019	13.23	11.25

### Checkerboard assay

Table 2 shows that two combinations of the peptides γ_33-41_*Pv*D_1_^++^ and AmB were able to generate additive interactions, and two combinations of the peptides γ_31-45_*Pv*D_1_^++^ and AmB were able to generate synergistic interactions. The additive interaction was shown at 0.009 µM γ_33-41_*Pv*D_1_^++^ and 13.23 µM AmB and with 0.019 µM γ_33-41_*Pv*D_1_^++^ and 6.61 µM AmB. The concentrations with synergistic interactions were 1.40 µM γ_31-45_*Pv*D_1_^++^ and 0.004 µM AmB and 0.70 µM γ_31-45_*Pv*D_1_^++^ and 0.002 µM AmB. From these data, we continue with the synergistic conditions in the action mechanism steps. The synergistic combinations identified in these studies demonstrate a difference when compared with the IC_50_ values of AmB and γ_31-45_*Pv*D_1_^++^. Specifically, the combination of 1.40 µM γ_31-45_*Pv*D_1_^++^ with 0.004 µM AmB concentrations was 4.5 and 8 times lower, respectively, than their individual IC_50_ values. For the combination of 0.70 µM γ_31-45_*Pv*D_1_^++^ and 0.002 µM AmB, the IC_50_ of AmB was 9.5-fold lower, and compared with the IC_50_ of the peptide γ_31-45_*Pv*D_1_^++^, it was 16-fold lower.

**Table 2:** Combined activity between bioinspired peptides and AmB

DRUG A	DRUG B	FIC A (µM)	FIC B (µM)	Σ FIC	Action
AmB	γ_33-41_PvD_1_^++^	0.009/0.019	13.23/13.23	1.47	Additive or Indifferent
	γ_33-41_PvD_1_^++^	0.019/0.019	6.61/13.23	1.49	Additive or Indifferent
	γ_31-45_PvD_1_^++^	0.004/0.019	1.40/11.25	0.33	Synergism
	γ_31-45_ PvD_1_^++^	0.002/0.019	0.70/11.25	0.16	Synergism

FIC values: ≤0.5 = Synergism; 0.5 < FIC < 1.0 = Additive or Indifferent; >1.0 = Antagonist.

### Viability assay

**Figure 2:**
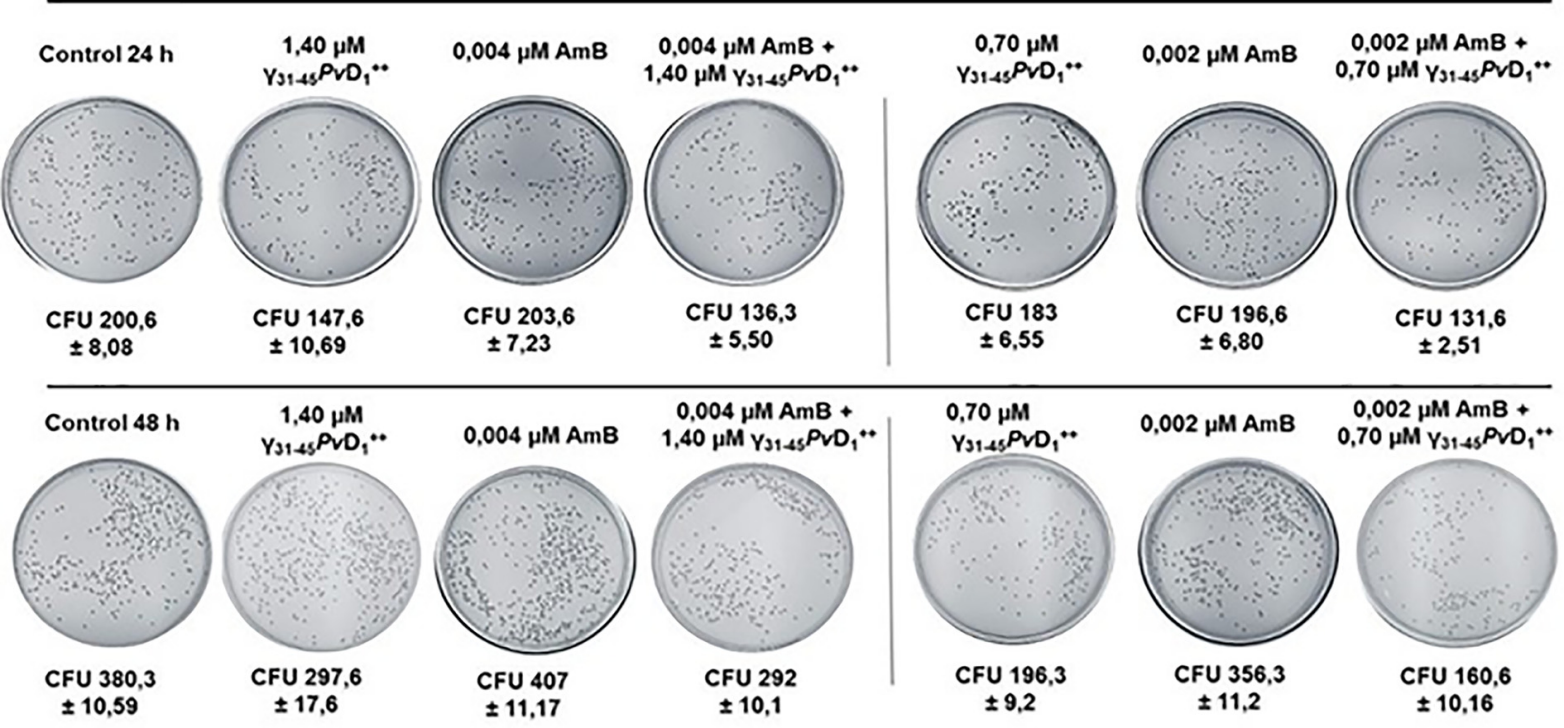
Growth of *C. albicans* cells incubated with peptides individually and in combination with AmB Growth of *C. albicans* yeast cells incubated with γ_31-45_*Pv*D_1_^++^ individually and in combination with AmB for 24 and 48 h. All experiments were performed in triplicate.

Figure 2 shows the cell viability assay. The synergistic combinations used individually were not able to cause a loss of viability when compared with the control. At 24 h of incubation, we observed the highest decline in viability in the combination of 0.70 µM peptide γ_31-45_*Pv*D_1_^++^ and 0.002 µM AmB, resulting in 65.60% viability. Additionally, the combination of 1.40 µM γ_31-45_*Pv*D_1_^++^ and 0.004 µM AmB peptides showed 67.9% viability (Table 3). After 48 h of testing, it was observed that *C. albicans* cells also exhibited susceptibility to the combination of 0.70 µM γ_31-45_*Pv*D_1_^++^ and 0.002 µM AmB peptides, resulting in an approximate viability of 42% (Table 3). These findings suggest that the inhibitory effect of the synergistic combinations is fungistatic.

**Table 3:** Viability of the *Candida albicans* cell culture after 24 h and 48 h of treatment with the synergistic conditions

Incubation time	Samples	CFU	Viability (%)	Loss of viability (%)
24h	Control	200.6	100	0
	1.40 µM γ_31-45_*Pv*D_1_^++^	147.6	73.57	26.43
	0.004 µM AmB	203.6	101,4	0
	0.004 µM AmB + 1.40 µM γ_31-45_*Pv*D_1_^++^	136.3	67.9	32.10
	0.70 µM γ_31-45_*Pv*D_1_^++^	183	91.22	8.78
	0.002 µM AmB	196.6	98	2
	0.002 µM AmB + 0.70 µM γ_31-45_*Pv*D_1_^++^	131.6	65.60	34.40
48h	Control	380.3	100	0
	1.40 µM γ_31-45_*Pv*D_1_^++^	297.6	78.2	21.80
	0.004 µM AmB	407	107.10	0
	0.004 µM AmB + 1.40 µM γ_31-45_*Pv*D_1_^++^	292	76	24
	0.70 µM γ_31-45_*Pv*D_1_^++^	196.3	51.56	48.39
	0.002 µM AmB	356.3	93	7
	0.002 µM AmB + 0.70 µM γ_31-45_*Pv*D_1_^++^	160.6	42.2	57.80

The CFU obtained in the control was assumed to be 100% of viability.

### Analysis of mitochondrial functionality

**Figure 3:**
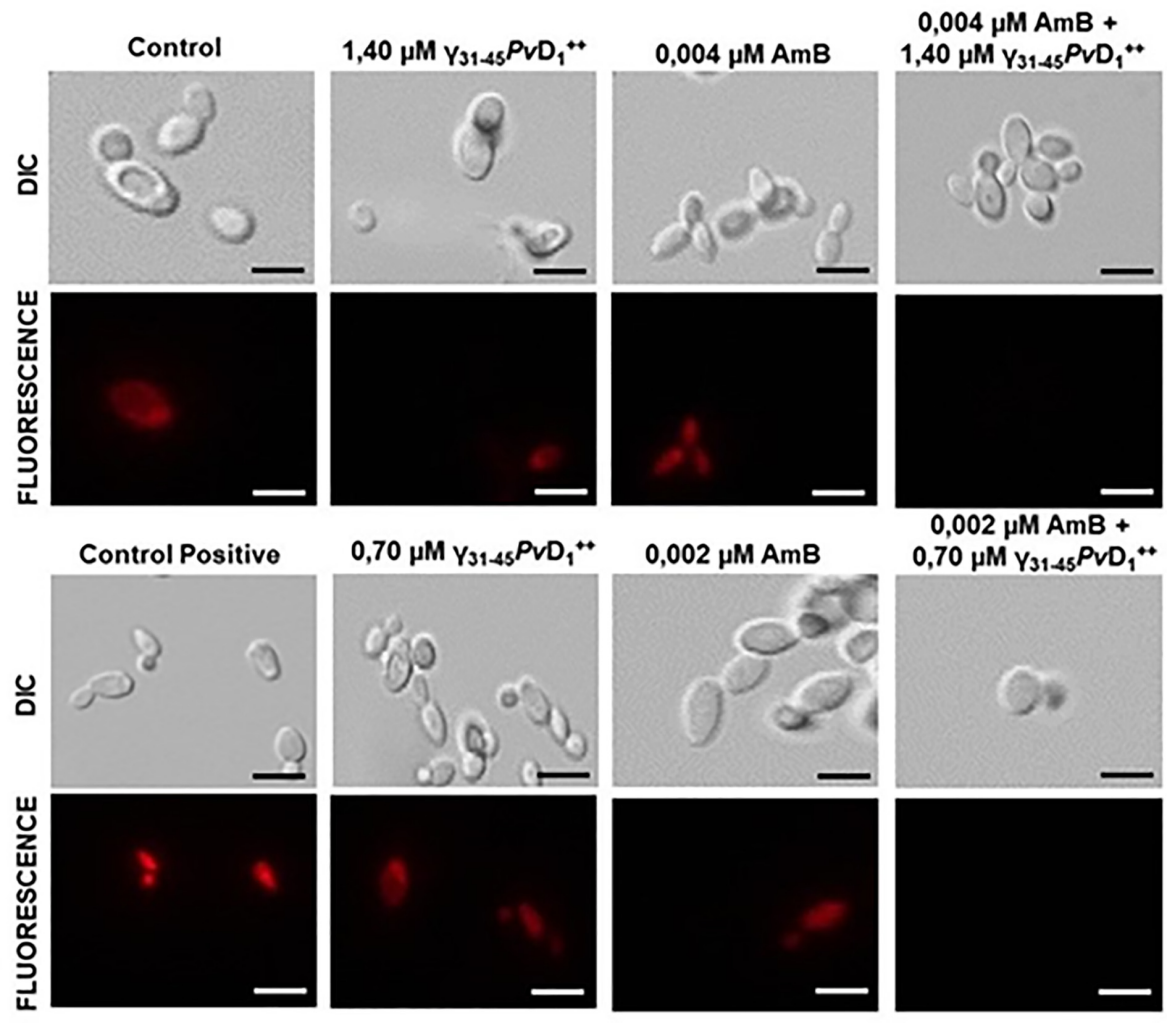
Mitochondrial functionality assay of *C. albicans* cells Images of the mitochondrial functionality assay of *C. albicans* cells after treatment with γ_31-45_*Pv*D_1_^++^ individually and in combination with AmB for 24 h. The fluorescent probe MitoTracker was used to visualize mitochondrial activity. Positive control cells were treated with 300 mM acetic acid; bars = 20 µm.

The results demonstrate that the combination of synthetic peptide γ_31-45_*Pv*D_1_^++^ and AmB at synergistic concentrations leads to a loss of mitochondrial functionality in *C. albicans* cells (Figure 3). The synergistic concentrations of 1.40 µM γ_31-45_*Pv*D_1_^++^ and 0.004 µM AmB, as well as 0.70 µM γ_31-45_*Pv*D_1_^++^ and 0.002 µM AmB, induced a notable loss of mitochondrial functionality. Notably, when the peptides or AmB were used individually, at the same concentrations as the synergistic combination, the mitochondria maintained their functionality, similar to the controls.

### Chromatin condensation in *Candida albicans*

**Figure 4:**
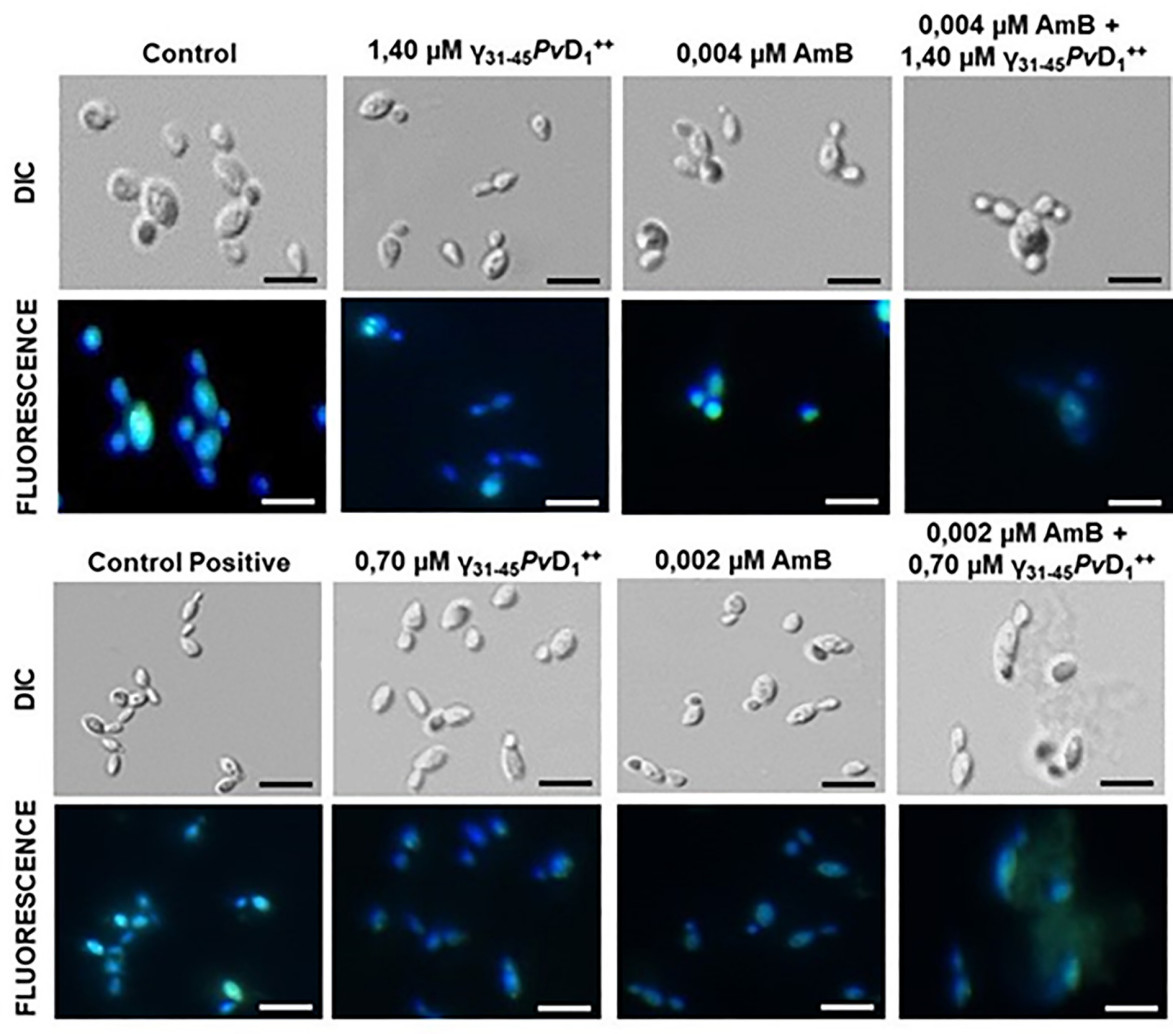
Chromatin condesation assay of *C. albicans* cells Chromatin condensation assay images of *C. albicans* cells after treatment with γ_31-45_*Pv*D_1_^++^ individually and in combination with AmB for 24 h. To visualize the nuclei, DAPI was used. As a positive control, cells were treated at 100°C for 1 min; bars = 20 µm.

As observed in Figure 4, the synergistic combinations of 1.40 µM γ_31-45_*Pv*D_1_^++^ and 0.004 µM AmB, as well as 0.70 µM γ_31-45_*Pv*D_1_^++^ and 0.002 µM AmB, caused damage to the DNA of *C. albicans*. In control cells, DAPI (4',6-diamidino-2-phenylindole) labeling exhibits intense fluorescence, indicating the intactness of cellular DNA. This characteristic is also evident in cells treated with individual concentrations of the synthetic peptide γ_31-45_*Pv*D_1_^++^ and AmB.

### Detection of wall integrity in *Candida albicans*

**Figure 5:**
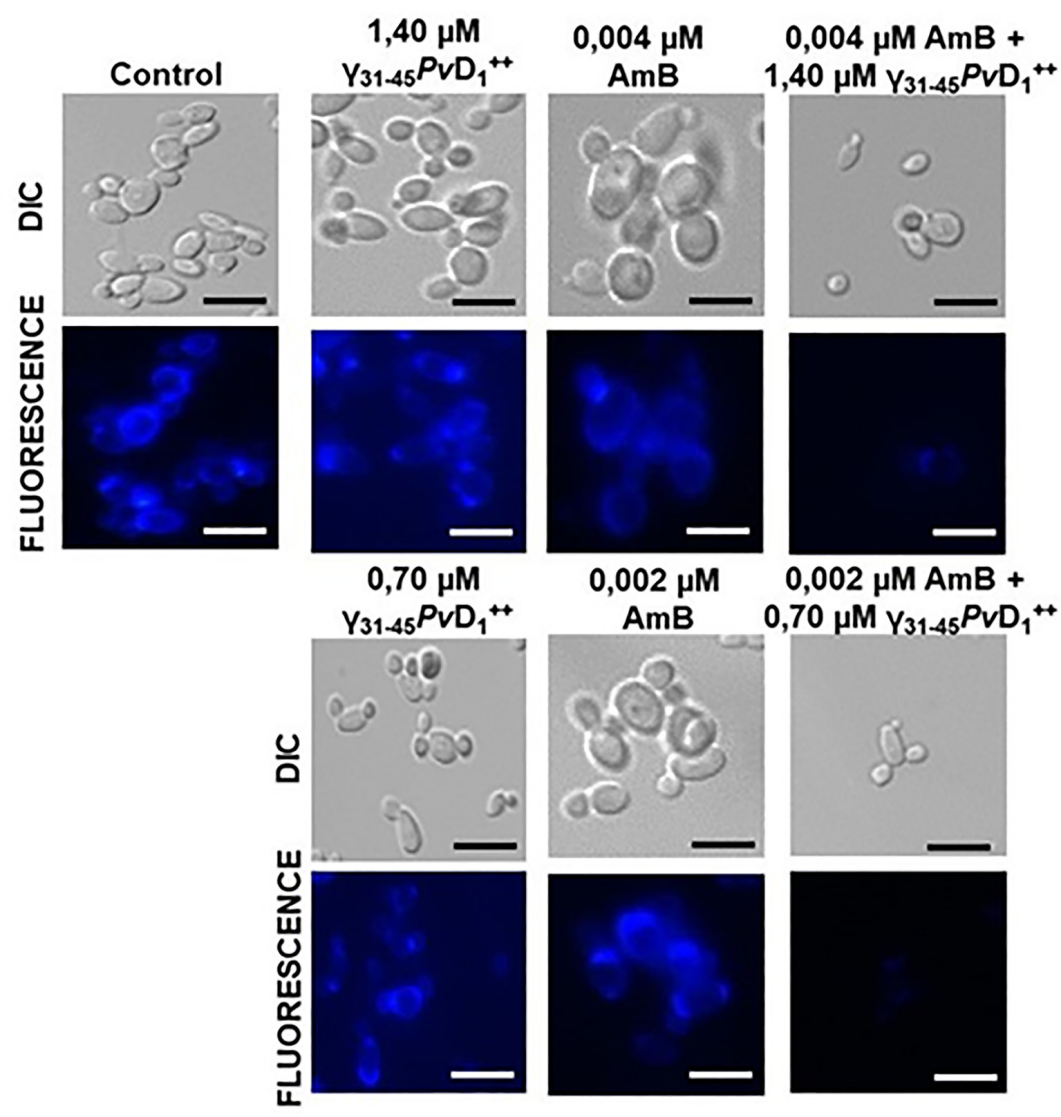
Cell wall integrity assay of *C. albicans* cells Image detection of the wall integrity of *C. albicans* cells after treatment with γ_31-45_*Pv*D_1_^++^ individually and in combination with AmB for 24 h. The wall integrity of *C. albicans* cells was assessed using a calcofluor white probe; bars = 20 µm.

We performed an analysis of the cell wall integrity of *C. albicans* following a 24 h assay in the presence of synergistic combinations of the synthetic peptides γ_31-45_*Pv*D_1_^++^ and AmB. In the control and individual concentrations of the peptide or AmB, we observed cells that were prominently stained with calcofluor White, indicating the integrity of their cell walls. The resulting blue fluorescence indicates the presence of an intact cell wall, as clearly depicted in Figure 5. However, after treatment with synergistic combinations, we no longer observed these intense cell markings but rather fragmented ones, suggesting the loss of cell wall integrity and possibly compromising cell viability.

### Ultrastructural alterations in *Candida albicans*

**Figure 6:**
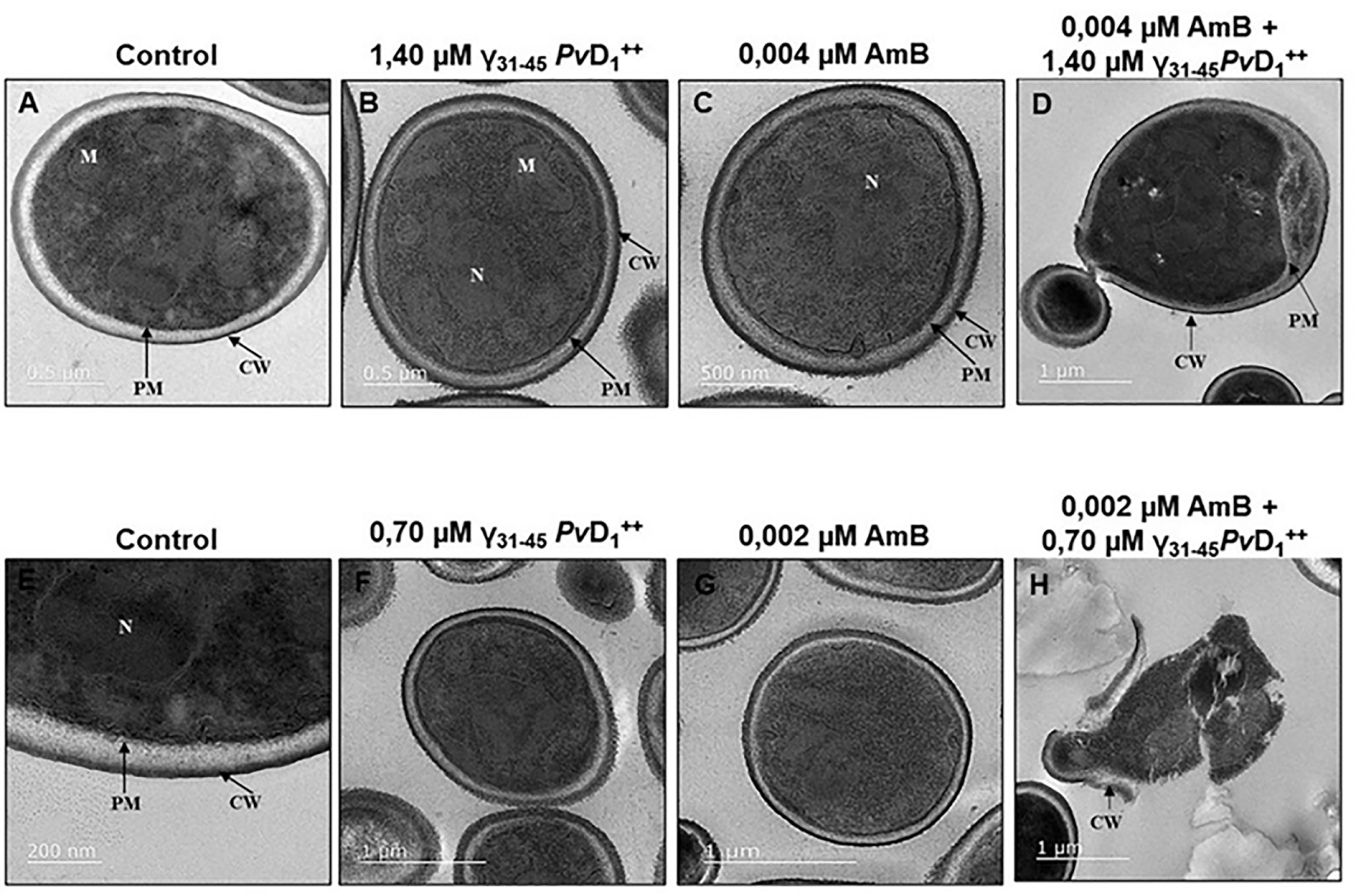
Transmission electron microscopy assay of *C. albicans* cells Ultrastructural changes visualized by transmission electron microscopy in *C. albicans* yeast cells after treatment with γ_31-45_*Pv*D_1_^++^ individually and in combination with AmB for 24 h. (**A**) negative control; (**B-C**) Individual γ_31-45_*Pv*D_1_^++^ and individual AmB respectively; (**F-G**) Individual γ_31-45_*Pv*D_1_^++^ and individual AmB respectively. In the synergistic combination between the peptide and AmB (**D, H**), the plasma membrane and cell wall are ruptured, which indicates cell permeabilization.

The ultrastructural features of *C. albicans* cells, encompassing all control treatments, are depicted in Figure 6. The most notable differences are observed within the cellular components of the cell wall and plasma membrane. The control cells presented a uniform surface without apparent rupture. However, under synergistic conditions, it is evident that the treated *C. albicans* cells show severe compromise of their plasma membrane and cell wall integrity. Additionally, in some cases, cell leakage is observed, and chromatin condensation is confirmed by TEM.

## Discussion

The escalating antimicrobial resistance to conventional drugs underscores the pressing demand for innovative and efficient alternatives. These alternatives should offer swift and comprehensive antifungal and antimicrobial action while minimizing potential safety-related impacts on infections [26].

In this work, we analyze the combination of synthetic peptides based on the region motif γ-core of a *V. unguiculata* seed defensin (*Pv*D_1_) and the commercially used drug AmB in developing *C. albicans*. The combination of synthetic peptides and conventional antifungals has increasingly become an alternative for producing a more efficient drug that is more efficient in low doses. In the synergism between these molecules, the combined action of the synthetic peptide with the same occurs, causing a potentiation in the decrease in the growth of the microorganism in comparison with the inhibition of the growth of the individual substances [16,18].

The γ-core region is a structural element shared by most host antimicrobial peptides (AMPs), which in some AMP families, such as defensins, contribute to their antimicrobial properties. This motif has been extensively studied in the search for new anti-infective agents [19].

We started by determining the minimum inhibitory concentration (MIC_50_) of synthetic peptides and AmB against *C. albicans*. The MIC_50_ values for γ_33-41_*Pv*D_1_^++^, γ_31-45_*Pv*D_1_^++^, and AmB were 13.23, 11.25, and 0.019 µM, respectively (Table 1 and Figure 1). Fungal cells employ various defense mechanisms to counteract the toxicity of AmB. The cell wall immobilizes AmB molecules, and extracellular structures are binding sites for these molecules. Recent studies have shown the specific biological activity of AmB in *C. albicans* cells, particularly its affinity for binding to the cell membrane of young cells during the budding stage. This interaction significantly affects the membrane’s structural properties and the transport of physiological ions and penetration into the cytoplasm. Consequently, various intracellular organelles and physiological processes are affected as a result [27].

Some mechanisms of action of antimicrobial peptides from plants against yeast have been elucidated, especially in the case of defensins, such as plasma membrane permeabilization, mitochondrial damage and DNA damage, impairing the cell cycle [28]. Therefore, we analyzed whether some of these events were capable of affecting the yeast *C. albicans* when treated with the synergism between AmB and the synthetic peptide.

To assess synergistic combinations, a checkerboard assay was employed. This assay evaluates the impact of a test substance in combination with an antimicrobial agent at various concentrations. Our results demonstrate two combinations with additive effects and two with synergistic effects (Table 2).

In 2023 [29] the lead compounds LP-23, DP-23, SA4, and SPO, synthesized via solid-phase peptide synthesis, exhibited synergistic effects when combined with specific antibiotics. This synergy was observed against a spectrum of bacterial and fungal strains, underscoring the potential of these compounds in enhancing antimicrobial efficacy. The assessment of peptide-antibiotic combinations involved the utilization of the checkerboard method. The minimum inhibitory concentration (MIC) in these combinations was expressed using the fractional inhibitory concentration index (FIC). Virtually all pairings of peptides or with AmB and fluconazole exhibited potent synergy against *Aspergillus niger* and *Aspergillus flavus*. For instance, the FIC index of combinations such as LP-23 + AmB, DP-23 + AmB, SA4 + AmB, and SPO + AmB were 0.141 and 0.275, 0.290 and 0.267, 0.321 and 0.251, and 0.385 and 0.186, respectively. These findings underscore the potential of these combinations in combating these fungal strains effectively. In a recent review conducted by [14], the authors expound upon a series of synergistic peptide combinations with AmB, exhibiting efficacy against *Candida* spp. noteworthy examples encompass the peptide combination LL-37 + AmB, revealing FIC values ranging between 0.13 and 0.31 against *Candida auris*. Another finding entails the peptide combination Dq-3162 + AmB, showcasing an FIC value of 0.3125 for *C. albicans*, *C. tropicalis*, and *C. krusei*. Similarly, the interaction involving peptide Dq-2562 + AmB exhibited an FIC value of 0.3125 against the same strains: *C. albicans*, *C. tropicalis*, and *C. krusei*. These observations underscore the potential of these combined approaches in tackling these specific *Candida* strains effectively. In our study, we discovered FIC values that closely paralleled those reported in the aforementioned review. Specifically, our investigation revealed synergistic peptide and AmB combinations that yielded FIC values of 0.16 and 0.33 against *C. albicans*. In 2020 [2], made a significant discovery, identifying a synergistic effect between AmB and several drugs, including erythromycin, riluzole, nortriptyline, kenodiol, nisoldipine, promazine, chlorcyclizine, cloperastine, and glimepiride. Notably, all interactions with AmB demonstrated a synergistic effect, as evidenced by a low inhibitory concentration (FIC index of 0.5). This synergistic action was particularly observed in the inhibition of *C. albicans* spp. and *Cryptococcus neoformans*, highlighting the potential of this drug combination for therapeutic applications against these pathogens.

Among the mechanisms of action already described for peptides with antimicrobial activity, the interaction with the target membrane is one of the most studied. This interaction can result in pore formation or a direct interaction with a lipid domain, causing membrane permeabilization. Therefore, this mechanism of action makes antimicrobial peptides good models for understanding the mechanisms that lead to microorganism death. MitoTracker Red, a nontoxic fluorescent chemical probe containing a thiol chloromethyl-reactive fraction, is employed to visualize the distribution of mitochondria in living cells over an extended period. Additionally, it enables the quantification of mitochondrial potential through fluorescence microscopy analysis, as described by [30]. We analyzed whether synergistic combinations were capable of causing a loss of mitochondrial functionality in *C. albicans* cells, where the combinations were capable of causing disturbances in mitochondrial functionality within 24 h of the test (Figure 3).

Analyzing mitochondrial functionality serves as an effective method for investigating the mechanism of action of therapeutic molecules, including synthetic peptides. In a recent study [31], this approach was employed to examine the impact of a synthetic peptide derived from a plant defensin. The study revealed that the peptides RR (27.5 µM) and D-RR (23 µM) induced a rapid loss of mitochondrial functionality within just 1 and 6 h of incubation, respectively, in *C. tropicalis* cells. Similarly, in the case of *C. albicans*, the synthetic peptide WR demonstrated the ability to cause a comparable effect within 1 h of incubation.

Initially, it was believed that the mechanism of action responsible for the inhibition or killing of microbial growth by antimicrobial peptides involved damage to the microbial cell membrane. In fact, it is known that some antimicrobial peptides are able to interact with components of the microbial membrane, such as phospholipids and sphingolipids, and are involved in some cellular signaling cascades that involve mitochondrial functioning and the integrity of nucleic acids [32].

In addition to the observed loss of mitochondrial functionality, our study also explored the impact of synergistic combinations on other organelles and the cell wall. Particularly noteworthy was the detection of concentrated and intense nuclear fluorescence in both the control group and individual concentrations of the synergistic combinations. This finding strongly indicates the preservation of nuclear integrity, as illustrated in Figure 4. In contrast, the absence of these nuclear markings in the case of the synergistic combinations strongly suggests significant chromatin condensation or the potential leakage of DNA along with intracellular materials. These observations indicate a cell wall and plasma membrane disruption, leading to compromised cellular integrity. To assess nuclear morphology and DNA integrity in cells, we utilized DAPI. This cell-nonpermeable DNA-binding substance selectively interacts with the minor groove of double-stranded DNA, resulting in fluorescence emission. By specifically binding to DNA, DAPI facilitates the visualization and evaluation of cellular processes involving DNA, such as chromatin condensation and potential leakage of genetic material caused by cell wall and plasma membrane disruptions. The utilization of DAPI as a tool in our study proved valuable in understanding the underlying cellular changes induced by the synergistic combinations [22]

The cell wall is a cell structure found in various organisms, including fungi, plants, and bacteria, regardless of whether it is eukaryotic or prokaryotic. It serves multiple critical functions, contributing to the overall health and viability of the cell. Its primary role is maintaining cell integrity and shape, providing structural support, and preventing cell collapse. Additionally, the cell wall acts as a protective barrier for the plasma membrane, shielding the cell from external stresses and potential damage [33]. In the present study, we successfully demonstrated that both synergistic combinations caused significant damage to the cell wall of *C. albicans* cells (Figure 5). The control cells and the individual components of the synergistic combinations displayed intact cell walls with marked fluorescence. In the cells treated with the synergistic combinations, fragmented fluorescent labeling was evident, strongly suggesting a loss of integrity in the cell wall of *C. albicans*. These findings hold significant promise for developing novel antifungal therapies. Understanding how these synergistic combinations bring about such pronounced effects on the cell wall can offer valuable insights into the mechanisms of action and aid in designing more targeted and effective treatments against fungal infections.

The investigation of peptides with antimicrobial activity targeting the cell wall of microorganisms has captivated the attention of numerous scientists seeking innovative therapeutic alternatives. In recent years, the rise of antibiotic-resistant pathogens has posed significant challenges in treating infectious diseases. Therefore, exploring alternative antimicrobial agents, such as peptides, has become a priority in biomedical research [34].

In 2023, Reis [35] made a significant discovery by identifying a potent host defense mimetic peptide called brilacidin (BRI). Their findings revealed that when used in combination with CAS, BRI exhibited remarkable synergistic effects against both CAS-sensitive and CAS-resistant isolates of various pathogenic fungi, including *Aspergillus fumigatus*, *C. albicans*, *C. auris*, and *Cryptococcus neoformans*, the latter being intrinsically resistant to CAS. The mode of action of BRI involves two critical mechanisms, enabling its impressive antifungal activity. First, BRI effectively affects the cell wall integrity pathway of fungal pathogens, destabilizing their cell walls. This disruption severely compromised the structural integrity of the fungal cells, making them more susceptible to further treatment by antifungal agents such as CAS.

The present study observed the impact of synergistic combinations on the morphology and ultrastructure of *C. albicans* (Figure 6). The TEM images reveal a notable difference in the structure of the plasma membrane and cell wall, thus supporting the data presented thus far in the present study. The plasma membrane and cell wall appear intact in the control cells and the individual concentrations of the synergistic combination. However, in the case of the synergistic combinations, a distinct alteration in the plasma membrane and cell wall morphology becomes evident, underscoring the disruptive effects of the combination treatment on these vital cellular components. The synergistic combinations manifest profound effects on *C. albicans* cells, as evidenced by the detachment of the plasma membrane and cell wall, abnormal cell morphology, and disruption of cytoplasmic content and organelles. These observations strongly suggest that the anti-*Candida* activity of the synergistic combinations primarily relies on their impact on membrane permeability and the cell wall. This approach targets various cellular components, including mitochondria and the nucleus, which aligns with the well-established antifungal mode of action exhibited by polyenes such as nystatin and AmB. The observed deformities in *C. albicans* cells provide further support for the disruptive effects of the synergistic combinations, leading to the impairment of essential cellular processes and ultimately inhibiting *Candida* growth.

In 2020 [36], Seyedjavadi et al. focused on the mode of action of the synthetic peptide MCh-AMP1 against the growth of *C. albicans*. Their study revealed similar outcomes, illustrating the peptide’s impact on the cellular mechanisms of *C. albicans*. The research demonstrated that MCh-AMP1 targeted the membrane integrity of *C. albicans*, leading to membrane disruption, altered cell morphology, and impaired organelle function. These findings further support the notion that antimicrobial peptides, such as MCh-AMP1 and our synergistic combinations, exert their antifungal effects through multiple pathways, including membrane destabilization and disruption of vital cellular components. In 2016, Yu et al. [33] highlights the fungicidal effect of the synthetic peptide MCh-AMP1 at 32 and 64 µg/ml concentrations. The peptide targeted disrupted membrane integrity, resulting in increased permeability. Additionally, the study revealed that MCh-AMP1 treatment led to the generation of reactive oxygen species (ROS), further contributing to antimicrobial action. These findings provide valuable insights into the mode of action of MCh-AMP1, underscoring its ability to effectively combat fungal growth by targeting critical cellular components such as the membrane and inducing oxidative stress through ROS production.

In the face of an escalating global menace posed by antibiotic resistance, antimicrobial peptides (AMPs) have emerged as a promising new class of drugs in the fight against diverse infectious diseases. The 2023 [37] Safronova et al. shed light on the compelling potential of AMPs as a viable alternative to traditional antibiotics. The heightened interest in AMPs is particularly noteworthy, driven by recent outbreaks of secondary infections during the COVID-19 pandemic, which have exacerbated the urgent need for effective agents against bacterial and fungal infections. This comprehensive review serves as a timely reminder of the critical role AMPs can play in combating infectious diseases, especially when conventional antibiotics struggle against drug-resistant pathogens. With the acute shortage of suitable therapeutic options, the exploration of AMPs represents a promising avenue in the search for innovative treatments.

## Conclusion

This discovery reveals the antifungal effect of synergistic combinations of the synthetic peptide γ_31-45_*Pv*D_1_^++^ and AmB against the yeast *C. albicans*. This combination targets the plasma membrane and cell wall. In addition, this synergism is also capable of causing loss of cellular functionality and DNA degradation. Together, these data represent a promising new therapeutic option for the treatment of fungal diseases associated with this microorganism.

## Data Availability

The data that support the findings of this study are available from the corresponding author upon reasonable request.

## Abbreviations

AmB: amphotericin B
AMP: antimicrobial peptide
BRI: brilacidin
CAS: caspofungin
CFU: colony forming units
CW: cell wall
DIC: differential interference contrast
M: mitochondria
N: nucleus
PM: plasma membrane
